# Establishment of Streptococcus suis Biofilm Infection Model *In Vivo* and Comparative Analysis of Gene Expression Profiles between *In Vivo* and *In Vitro* Biofilms

**DOI:** 10.1128/spectrum.02686-22

**Published:** 2022-12-12

**Authors:** Li Yi, Qingying Fan, Haikun Wang, Haoran Fan, Jing Zuo, Yuxin Wang, Yang Wang

**Affiliations:** a College of Life Science, Luoyang Normal University, Luoyang, China; b College of Animal Science and Technology, Henan University of Science and Technology, Luoyang, China; c Key Laboratory of Molecular Pathogen and Immunology of Animal of Luoyang, Luoyang, China; Ohio State University

**Keywords:** *Streptococcus suis*, biofilm, selective capture of transcribed sequences, *in vitro*, *in vivo*

## Abstract

Streptococcus suis is a zoonotic pathogen that continuously threatens animal husbandry and public health worldwide. Studies have shown that S. suis can cause persistent infection by forming biofilms. In this study, a model of S. suis biofilm-related infection was successfully constructed for the first time by simulating the natural infection of S. suis, and biofilm of S. suis *in vivo* was successfully observed in the lung tissue of infected pigs by a variety of detection methods. Subsequently, selective capture of transcribed sequences (SCOTS) was used to identify genes expressed by S. suis *in vivo* biofilms. Sixty-nine genes were captured in *in vivo* biofilms formed by S. suis for the first time by SCOTS; they were mainly involved in metabolism, cell replication, and division, transport, signal transduction, cell wall, etc. Genes related to S. suis *in vitro* biofilm formation were also identified by SCOTS and RNA sequencing. Approximately half of the genes captured by SCOTS in the *in vivo* and *in vitro* biofilms were found to be different. In summary, our study provides powerful clues for future exploration of the mechanisms of S. suis biofilm formation.

**IMPORTANCE** Streptococcus suis is considered an important zoonotic pathogen, and persistent infection caused by biofilm is currently considered to be the reason why S. suis is difficult to control in swine. However, to date, a model of the biofilm of S. suis *in vivo* has not been successfully constructed. Here, we successfully detected biofilms of S. suis *in vivo* in lung tissues of piglets infected with S. suis. Selective capture of transcribed sequences and the transcriptome were used to obtain gene profiles of S. suis *in vivo* and *in vitro* biofilms, and the results showed large differences between them. Such data are of importance for future experimental studies exploring the mechanism of biofilm formation by S. suis *in vivo*.

## INTRODUCTION

Streptococcus suis is an important zoonotic pathogen, and strains represented by serotype 2 continue to threaten public health security worldwide (1, 2). Pigs are generally considered to be the natural host of S. suis and are mainly colonized in areas of the upper respiratory tract, such as the tonsils and the nasal cavity, and S. suis generally does not cause disease in pigs. Pigs acutely infected with S. suis often show meningitis, arthritis, septicemia, and even death (3). Exposure to infected animals and their products is often the key route of transmission of human infection by S. suis, so this pathogen has always been a focus of research in the field of veterinary and human medicine. The current form of prevention and control of S. suis is still not optimal. The reasons are that, on one hand, vaccines would be an ideal way to control the spread of S. suis infections, but there is no commercial vaccine to prevent and control S. suis (4), and on the other hand, to a large extent, persistent infections result in spreading of S. suis, causing great difficulties in the prevention and control of this disease. Biofilm formation largely explains the cause of persistent infections, and S. suis has been shown to cause persistent infections by forming biofilms that in turn cause bacterial resistance to antibiotics and increase the probability of horizontal transmission of antibiotic resistance genes (2).

Actually, there is no lack of research on the regulatory mechanism of S. suis biofilm formation. In the early stage, our research team carried out a series of explorations on the mechanisms of formation of S. suis biofilm and the influence of biofilm on the virulence and antibiotic resistance of S. suis (2, 5, 6). It is worth noting that most of the previous studies on S. suis biofilms relied on *in vitro* models. In fact, most of the current research on biofilms relies on *in vitro* culture models, such as 24- and 96-well microtiter plates and dynamic biofilm culture models represented by parallel-plate flow chambers, CDC biofilm reactors, and drip flow reactors (DFRs) (7). Although the dynamic model can better simulate the fluidity in the host environment and the shear force generated when liquid flows and is closer to the conditions in the living environment than the static model, it is not widely used by researchers due to its high price. In contrast, the static model based on the microtiter version is more favored by researchers because it is inexpensive and can meet the research needs of exploring the growth kinetics of biofilms and large-scale screening of antibiofilm drugs (8). Some investigators optimize *in vitro* biofilm models with natural substances like saliva or dental plaque or with synthetic materials using microcosm biofilm models in order to maximize the conditions for biofilm formation in the setting of reductive natural infections (9). Some studies sharply pointed out the disadvantages of the research based on the *in vitro* biofilm model (10, 11). Specifically, *in vitro* biofilms do not represent *in vivo* biofilms under real conditions. On one hand, the nutrient conditions that bacteria depend on to survive *in vitro* and *in vivo* are different; the nutrient-deficient conditions in the infection environment are different from the conditions in nutrient-sufficient medium (12). On the other hand, there is the absence of host-related biological factors under *in vitro* culture conditions, such as the surface properties of adhesion *in vivo*, the physicochemical characteristics and mechanical characteristics of the original microenvironment of the host *in vivo*, and the interference with and destruction of biofilm formation by host immune system components, such as immune cells, antibodies, and so on (13). No matter how well-established *in vitro* models are, there is no device that can mimic *in vivo* conditions in terms of adhesion surfaces, nutrient support, and microbial interactions. It follows that the indiscriminate imposition of research conclusions drawn from *in vitro* biofilm models into *in vivo* biofilm-related research is bound to misguide the research direction.

Based on previous findings by our group, S. suis can form biofilms in the muscle tissue of zebrafish, a model animal for S. suis (unpublished data). In addition, Ma et al. reported that biofilm S. suis was isolated from the liver, spleen, and kidney of healthy mice challenged with planktonic S. suis (14). A fair amount of evidence suggests that biofilm may be a real form of S. suis within the host. Unfortunately, however, an *in vivo* biofilm model of S. suis has not been successfully established to date. Therefore, the focus of research on the biofilm of S. suis should be placed on the biofilm *in vivo*, to better understand the mechanisms of biofilm formation of S. suis under real infection conditions and the interactions between the biofilm state bacteria and the host, so as to explore the key genes associated with S. suis biofilms. At present, three main types of *in vivo* biofilm models have been described, which are commensal biofilm models represented by the periodontal biofilm model, foreign-body models of biofilm infection represented by urinary tract infection and osteomyelitis models, and biofilm models of adherence to host mucosa or soft tissues (15, 16).

Selective capture of transcribed sequences (SCOTS) is a PCR-based RNA analysis method created by Graham and Clark-Curtiss in 1999 (17). It has been applied by different researchers to screen pathogenic bacteria for differential gene expression *in vivo*. This method can simply and quickly detect genes specifically expressed in living tissue samples under natural infection conditions and can also study the gene expression differences of bacteria under different stress conditions, such as low temperature, hypoxia, and anaerobicity (17–19). Isolation of low-abundance transcripts is a significant advantage of this technique, since each round of normalization enriches rare transcripts (20). SCOTS has been successfully applied in S. suis to capture the genes preferentially expressed upon interaction with porcine brain microvascular endothelial cells and under iron starvation conditions (19, 21). Researchers believe that SCOTS may be considered the only method that can directly explore global differential gene expression in S. suis (21). To establish an *in vivo* model of S. suis biofilm infection, this study simulated the natural form of infection by intranasal inoculation of S. suis to mimic chronic infection. Several methods, including hematoxylin and eosin (H&E) staining, scanning electron microscopy (SEM), immunohistochemistry, and immunofluorescence, collectively identified S. suis biofilm formation *in vivo*. Subsequently, SCOTS was used to capture the expression profiles of differentially expressed genes under S. suis *in vitro* and *in vivo* biofilm conditions, and the key genes involved in S. suis biofilm formation both *in vivo* and *in vitro* were successfully explored, which will provide new directions for research on the mechanisms of biofilm formation of S. suis *in vivo* and on therapeutic approaches against persistent infection caused by biofilm.

## RESULTS

### Establishment of S. suis biofilm *in vivo*: tissues with high bacterial loads are found in the lungs and spleen.

In our present pig model of S. suis chronic infection, typical symptoms appeared 48 to 72 h after infection. The results of Todd-Hewitt broth (THA) agar plate colony counting after 24 h, with CFU values higher in lungs and spleen than in other tissues, are shown in Fig. S1 in the supplemental material. Given that S. suis is a respiratory pathogen, it often persists in lungs, surviving and reproducing to cause pneumonia, which in turn leads to lung tissue lesions. In contrast, the spleen, as an important immune organ of the body, mainly plays an immune response in infectious diseases and filters foreign bodies and bacteria in the blood. Therefore, we selected the lungs of infected pigs for further study.

### *In vivo* biofilms of S. suis were detected in the lungs of artificially established chronically infected pigs.

**FIG 1:**
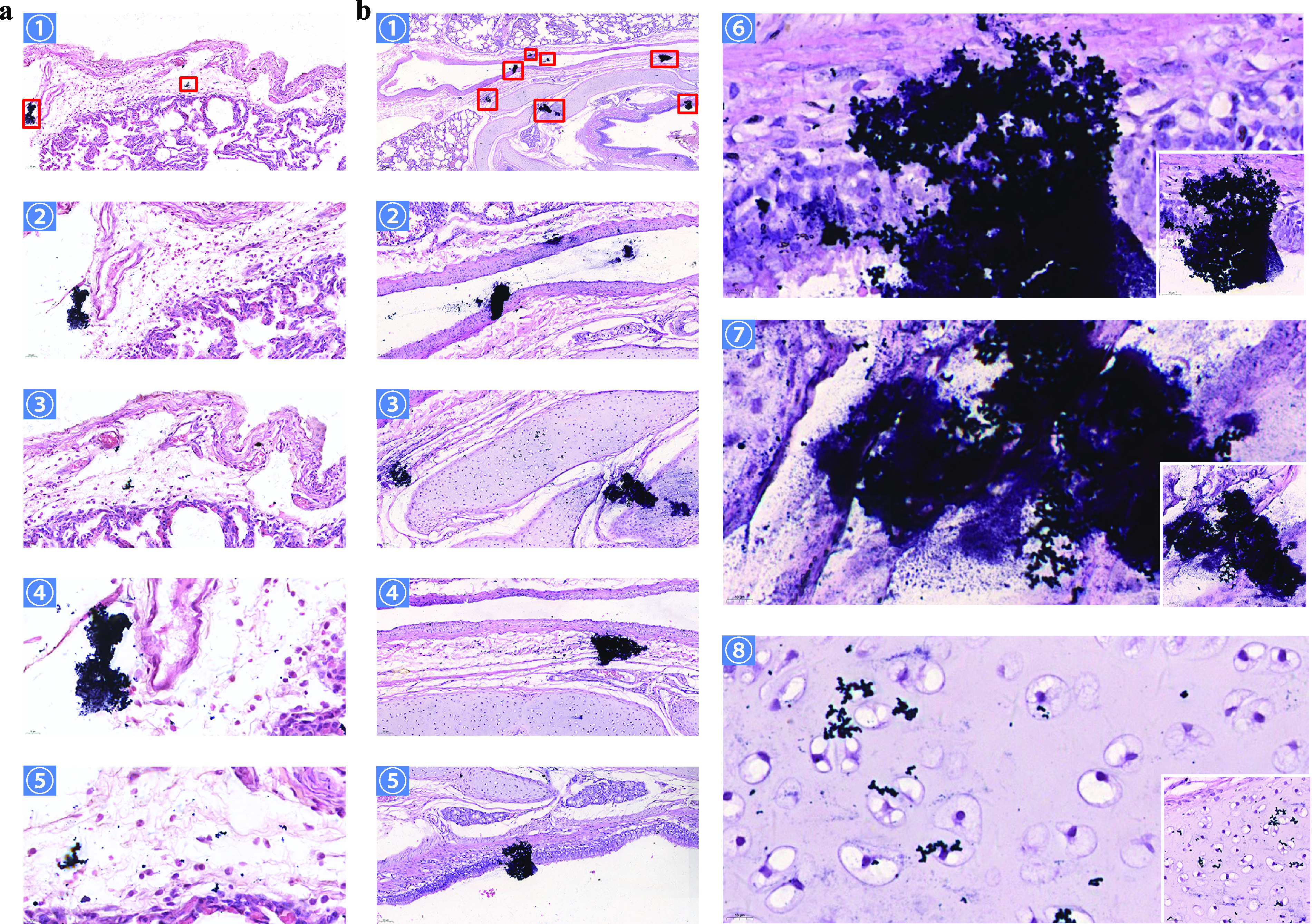
Hematoxylin and eosin (H&E) staining for detection of biofilms in lung tissues. (a) The images in panels 1 to 5 were made at ×200, ×400, ×400, ×1,000, and ×1,000 magnification, respectively. The images in panels 2 and 3 represent the ×400 magnification of the red solid box sections in panel 1, and similarly, the images in panels 4 and 5 represent the magnification of the images in panels 2 and 3. Basophilic aggregates of variable size are seen in the image in panel 1; the presence of chains of coccoid bacteria was discernible under ×1,000 magnification. (b) The images in panels 1 to 8 were made at ×50, ×200, ×200, ×200, ×200, ×1,000, ×1,000, and ×1,000 magnification, respectively. Seven basophilic aggregates of different sizes and shapes could be clearly found in the image in panel 1. Panels 2 to 5 represent the ×200 magnification of the red solid box in panel 1. Panels 6 and 7 represent the ×1,000 magnification of the images in panels 5 and 3, respectively. Panel 8 shows the magnified view of the basophilic granules scattered in the middle of the image in panel 3 observed at ×1,000 magnification. In the images in panels 6 and 7, chains of coccoid bacteria under basophilic aggregates can be clearly observed. Bacteria scattered in tissues can be further observed in panel 8. The bottom-right inset images in panels 6 to 8 are at ×600 magnification.

**FIG 2:**
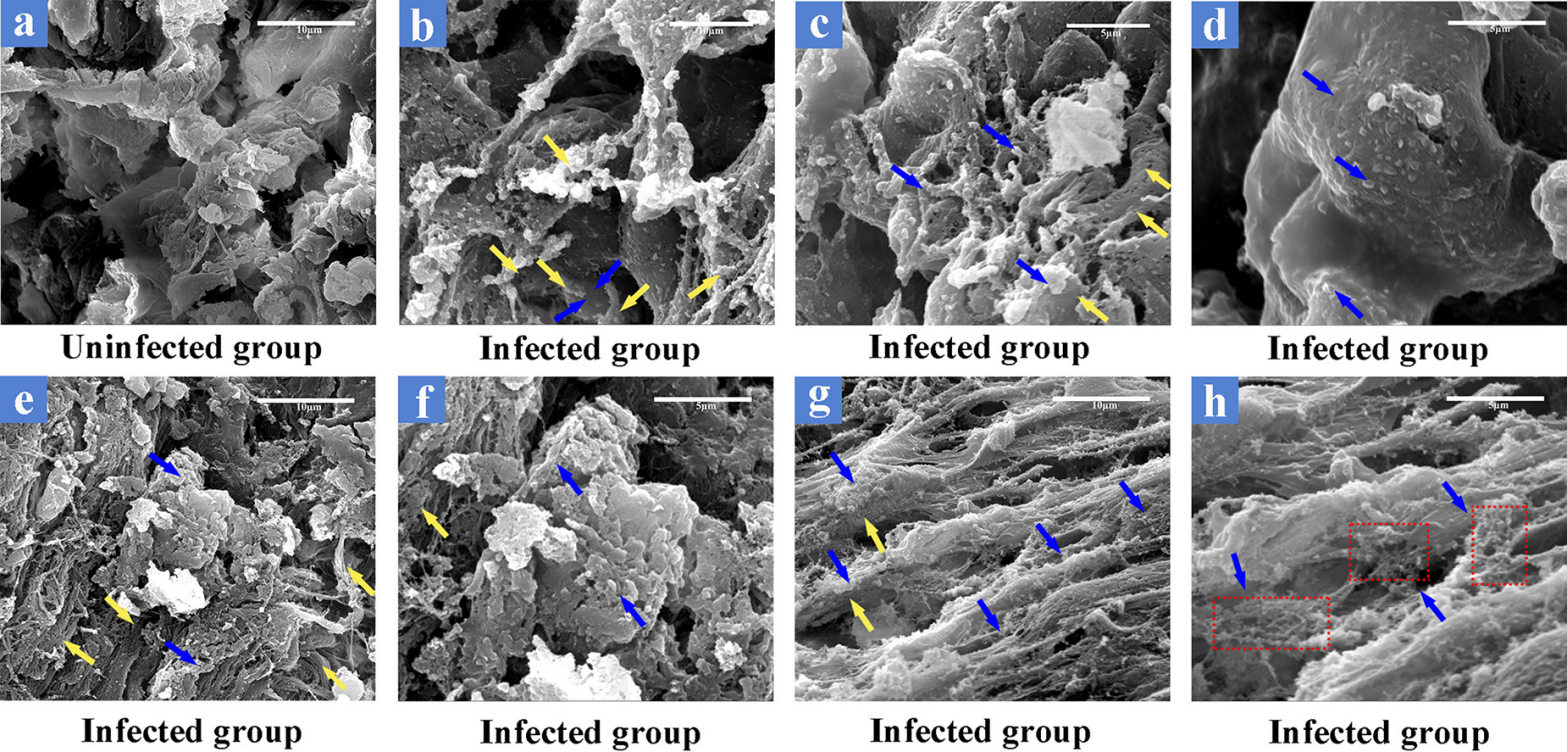
Scanning electron microscopy for detection of biofilms in lung tissues. (a) Image is representative of the lung tissue structure of uninfected piglets. (b to h) Images are representative of the lung tissue structure of infected piglets. In panels b and c, the structure of the host tissue and the cobweb-like biofilm matrix formed by extracellular polysaccharides of short chains of coccoid bacteria can be observed. In panel d, a dense matrix surrounds the bacteria to form a dense biofilm. Irregularly shaped dense material is present on ciliated tissue from the lung in panel e, and the presence of bacteria can be observed in panel f. In panel g, the surface of the lung ciliated tissue is covered with tiny cobweb-like structures carrying obvious bacteria. This structure is denser in the spaces between the ciliated tissues that are enclosed by the dashed boxes in panel h. Blue arrows represent the bacteria, as well as dehydrated extracellular matrix, and yellow arrows represent host tissue structures. The SEM images in panels a to h are at ×3,000, ×5,000, ×6,000, ×8,000, ×4,000, ×8,000, ×4,000, ×8,000 magnification, respectively. Scale bars represent 10 μm, 10 μm, 5 μm, 5 μm, 10 μm, 5 μm, 10 μm, and 5 μm, respectively.

In this study, four methods, including H&E staining, scanning electron microscopy, immunohistochemistry, and immunofluorescence, were used to identify the presence of biofilms of S. suis *in vivo*. Compared with those from the uninfected group (Fig. S2, left), the tissue sections from the S. suis-infected group (Fig. S2, right) showed obvious pathological changes, such as alveolar wall telangiectasia and congestion, as well as inflammatory cell infiltration, that were consistent with S. suis infection symptoms. Subsequently, we preliminarily detected the presence of biofilms of Streptococcus *in vivo* in some H&E-stained tissue sections from the infected group (Fig. 1). This study presents two groups of typical visual fields. For two different visual fields at low magnification, the presence of basophilic aggregates of various sizes and shapes was visible in both. With low magnification, the images in both Fig. 1a, panel 1, at ×200 magnification, and Fig. 1b, panel 1, at ×50 magnification, are representative, showing large or small aggregates mostly concentrated in loose connective tissue outside alveolar tissue, as well as in the adventitial layer (between airways and alveolar tissue), alveolar tissue, submucosa, mucosal layer, and even hyaline cartilage in the conductive zone of the lung. Under high magnification (×1,000), short chains of coccoid bacteria in basophilic aggregates can be clearly observed (compare Fig. 1a, panels 4 and 5 [×200], and Fig. 1b, panels 6 and 7 [×1,000]), especially in Fig. 1b, panel 7, where the matrix surrounding the bacteria shows a dispersed state. Interestingly, some basophilic aggregates appear to have dispersed. For example, in Fig. 1b, panels 2 and 3 (×200), the extracellular polymeric matrix that surrounds the bacteria appears to break down, with some bacteria dispersing to other parts of the tissue. Scattered bacteria can also be observed, such as in hyaline cartilage in Fig. 1b, panel 8 (×1,000). Based on the above-described results, we tentatively concluded that these aggregates were biofilms formed by streptococci in lung tissues. By evaluating lung sections using scanning electron microscopy, we found significant differences in the visual fields of infected and uninfected lungs. Relative to those of the uninfected group, the lung tissues in the infected group appeared to be covered with a layer of cobweb-like structures accompanied by the protrusion of fine particulate matter (Fig. 2b and c, g, and h). Blue arrows in Fig. 2 indicate the bacteria, as well as dehydrated extracellular matrix, and yellow arrows indicate host tissue structures. The cobweb-like structure comes from dehydration of the extracellular polymeric substance (EPS) of bacteria (Fig. 2, blue arrows) during sample preparation, with the coccoid bacteria wrapped within them to cover or attach to host tissue structures (Fig. 2, yellow arrows), and the presence of chain-like structures can be observed in some sites. Disordered lung ciliated tissue can be observed in Fig. 2e, and there is an irregular dense material on its surface. After local amplification of this part, spherical particles can be faintly seen exposed to the surface (Fig. 2f). Under another field of view (Fig. 2d), the dense matrix envelops the tightly arranged coccoid bacteria, completely covering the normal host tissue structure like a membrane structure, and there is an irregularly opened channel at the top.

**FIG 3:**
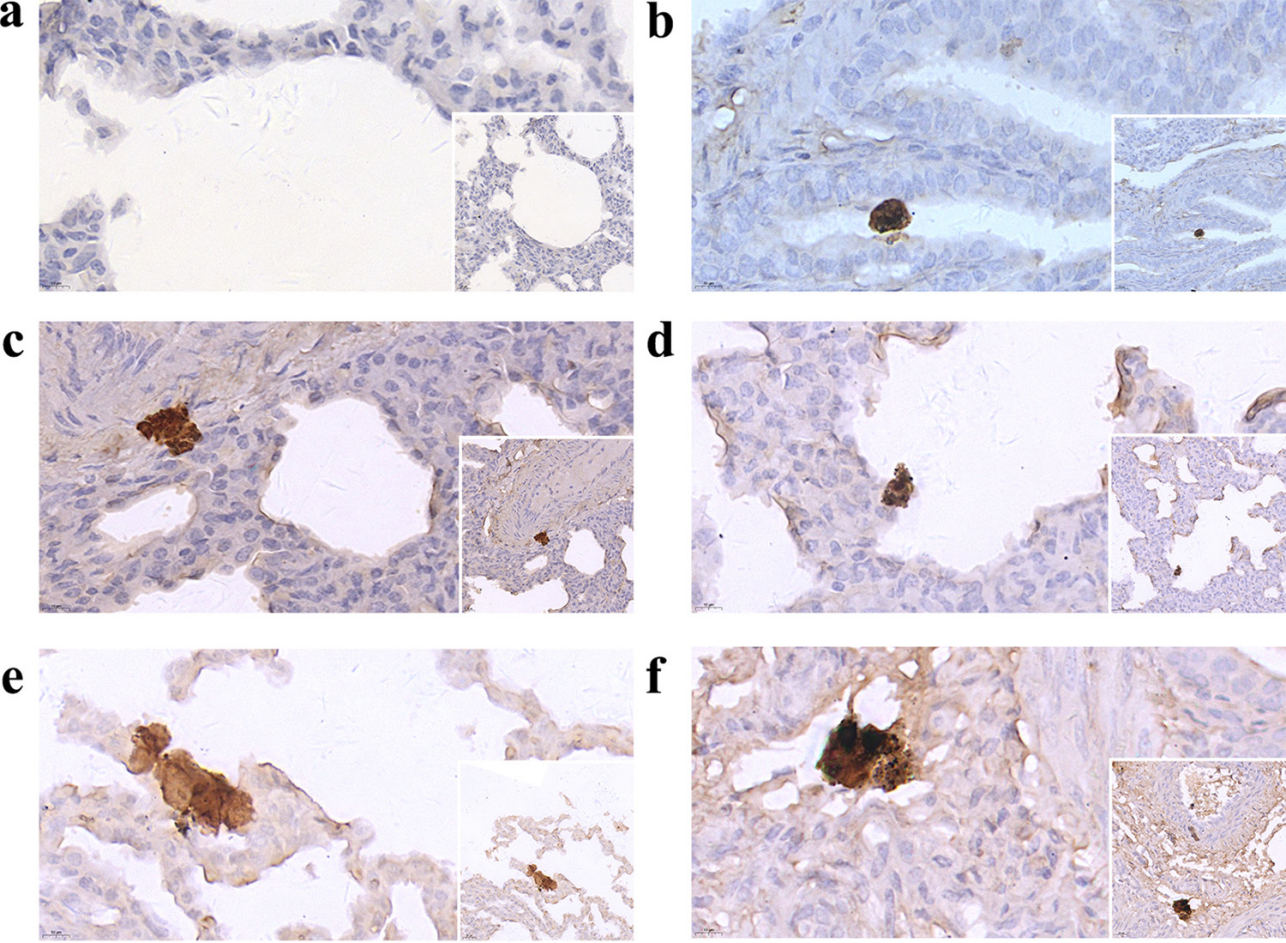
Detection of biofilms of S. suis in lung tissues by immunohistochemistry. (a) Image is representative of the lung tissues of uninfected piglets. (b to f) Images are representative of the lung tissues of infected piglets. Yellow or brown particle aggregates are detectable in the tissues of the infected piglets. Main panels, ×1,000 magnification; bottom-right insets, ×600 magnification.

**FIG 4:**
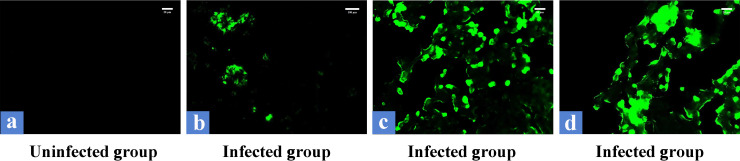
Detection of biofilms of S. suis in lung tissues by immunofluorescence. Clusters of green fluorescent signals are detected in the infected group, but not in the noninfected group. The images in panels a to d are at ×100, ×100, ×400, and ×400 magnification, respectively.

The structures described above are considered to be biofilms of Streptococcus *in vivo*. Based on the results of H&E staining and SEM, we confirmed that there were indeed *in vivo* biofilms in the lungs of pigs in the infected group. Given that the several methods described above did not confirm that the biofilms were formed by S. suis, immunohistochemistry and immunofluorescence were used for further exploration. The images in Fig. 3 indicate the distribution of biofilms of S. suis *in vivo* in lung tissues as shown by immunohistochemical detection of horseradish peroxidase (HRP)-labeled rabbit anti-pig IgG. Antigenic signals presenting as yellow or brown particle aggregates are clearly detectable in the tissues of the infected group (Fig. 3b to f) compared to those of the uninfected group (Fig. 3a) (Fig. 3, main panels, ×1,000, and bottom-right panels, ×600). In terms of the tissue sections in which positive aggregates are detected, aggregates of positive antigen vary in size and morphology and are mostly found in nonrespiratory zones of lung tissues (Fig. 3b, c, e, and f), while there is relatively little chance of detecting the presence of positive antigen aggregates in the alveolar areas (Fig. 3d), which is compatible with our results in H&E staining. Finally, the immunofluorescence results show green fluorescence clusters in the infected group and no green fluorescence signal in the uninfected group under dark-field conditions (Fig. 4). At ×100 magnification, two large clusters of irregularly shaped green fluorescent clusters in the field of view, as well as some scattered, distributed green fluorescent signals, were detectable (Fig. 4b). Under ×400 magnification, these two clusters of fluorescent signals presented much smaller fluorescent aggregates (Fig. 4c and d). From the results obtained, we can conclude that we found the existence of biofilm of S. suis *in vivo*.

### Selective capture of transcripts in the biofilm state of S. suis *in vitro* and *in vivo*.

**FIG 5:**
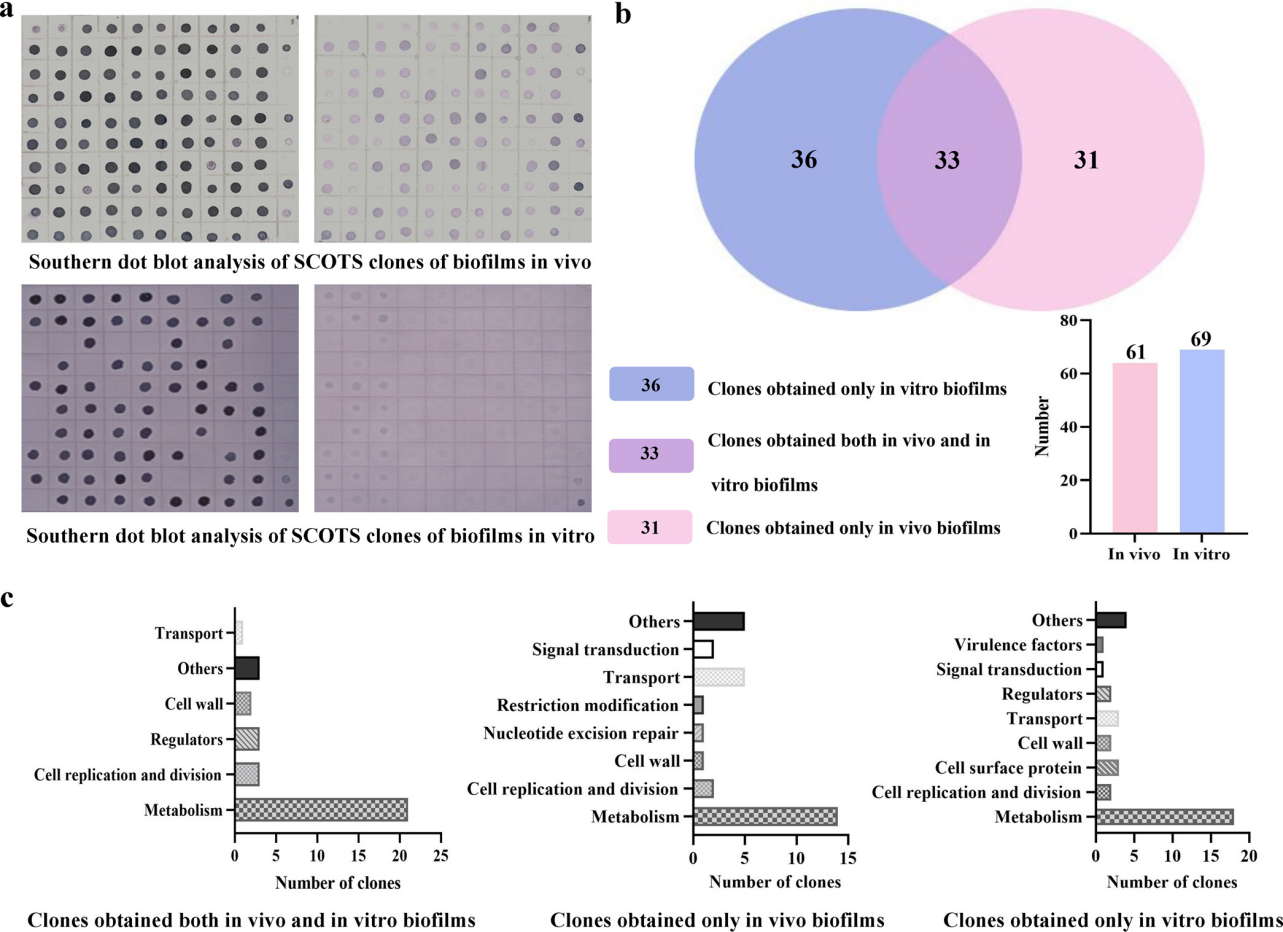
Selective capture of transcripts expressed in the biofilm state of S. suis *in vitro* and *in vivo*. (a) Southern dot blot analysis of SCOTS clones of biofilms *in vivo* and *in vitro*. (b) Results of SCOTS clones. A total of 61 positive clones were captured in *in vivo* biofilms, while 69 clones were captured in *in vitro* biofilms; 33 clones were cocaptured in *in vitro* and *in vivo* biofilms, 36 clones were specifically captured in *in vitro* biofilms, and 31 clones were specifically captured in *in vivo* biofilms. (c) Functional classification of clones captured by SCOTS. From left to right are cocaptured clones in *in vivo* and *in vitro* biofilms, clones specifically captured in *in vivo* biofilms, and clones specifically captured in *in vitro* biofilms.

After two rounds of double-dot hybridization validation, 64 genes and 69 genes were detected in the *in vivo* and *in vitro* biofilms, respectively (Fig. 5a). Among them, 33 genes were detected in both *in vivo* and *in vitro* biofilm states. Specifically, 21 genes were related to bacterial metabolism, involving carbohydrate metabolism, amino acid metabolism, nucleotide metabolism, energy metabolism, protein synthesis and hydrolysis, 3 genes were involved in cell replication and division, 3 genes encoded bacterial regulators, 2 genes were involved in bacterial cell wall synthesis, 1 gene was involved in transport, and 3 genes had unknown functions (Fig. 5b and Table 1). Excluding genes that were detected in both *in vitro* and *in vivo* biofilms, 31 genes were specifically detected under *in vivo* biofilm conditions, of which 14 genes were involved in bacterial metabolism, including carbohydrate metabolism, amino acid metabolism, nucleotide metabolism and synthesis, amino sugars and amino sugar and nucleotide sugar metabolism, and protein synthesis and hydrolysis, capsular polysaccharide synthesis, 5 genes were involved in transport, 2 genes were involved in bacterial signal transduction, 1 gene was involved in bacterial restriction modification, 1 gene was involved in bacterial nucleotide excision repair, 1 gene was involved in bacterial cell wall synthesis, and 5 genes were of unknown classification (Fig. 5c and Table 2). There were 36 genes specifically expressed under *in vitro* biofilm conditions, of which 18 genes were related to bacterial metabolism, involving carbohydrate metabolism, amino acid synthesis and metabolism, nucleotide metabolism, energy metabolism, protein biosynthesis and hydrolysis, ribosome synthesis, and cellular redox processes, 2 genes were involved in the replication and division of bacteria, 2 genes were involved in the synthesis of bacterial cell wall, 2 genes encoded cell surface proteins, 3 genes were involved in transport, 1 gene was involved in bacterial signal transduction, 2 genes were involved with transcriptional regulators, 1 gene encoded a virulence factor, and 4 genes had unknown classification (Fig. 5c and Table 3).

**TABLE 1:** Genes transcribed under both *in vivo* and *in vitro* biofilm conditions of S. suis captured by SCOTS

Category	Locus tag	Protein accession no.	Putative function
Carbohydrate metabolism	ZY05719_00790	AKG39571.1	Type II secretory pathway, pullulanase PulA and related glycosidases
	ZY05719_01910	AKG39782.1	4-α-Glucanotransferase
	ZY05719_09285	AKG41167.1	Dihydroxyacetone kinase
	ZY05719_03245	AKG40024.1	Phosphoglycerate dehydrogenase
	ZY05719_05855	AKG40512.1	Ribokinase family sugar kinase
Amino acid metabolism	ZY05719_09250	AKG41160.1	Leucyl aminopeptidase (aminopeptidase T)
	ZY05719_08880	AKG41090.1	Selenocysteine lyase
Nucleotide metabolism	ZY05719_10310	AKG41365.1	Inosine 5′-monophosphate dehydrogenase
	ZY05719_02630	AKG39910.1	Adenosine deaminase
	ZY05719_01840	None	Histone acetyltransferase HPA2-like acetyltransferase
Energy metabolism	ZY05719_00315	AKG39496.1	Multidrug ABC transporter ATPase
Protein biosynthesis	ZY05719_00465; *rplV*	AKG39524.1	50S ribosomal protein L22
	ZY05719_03945; *trmD*	AKG40148.1	tRNA [guanine-*N* (1)-]-methyltransferase
	ZY05719_10065; *argS*	AKG41316.1	Arginyl-tRNA synthetase
	ZY05719_09220	AKG41154.1	Translation factor (SUA5)
	ZY05719_09240	AKG41158.1	2-Isopropylmalate synthase
	ZY05719_06080	AKG40557.1	30S ribosomal protein S16
Proteolysis	ZY05719_08140	AKG40947.1	Cysteine aminopeptidase C
	ZY05719_09405	AKG41190.1	Subtilisin-like serine protease
	ZY05719_02485	AKG39882.1	Collagenase-like protease
	ZY05719_00870	AKG39587.1	Chaperonin GroEL
Cell replication and division	ZY05719_00565	AKG39544.1	DNA-directed RNA polymerase subunit α
	ZY05719_06580	AKG40654.1	DNA polymerase I
	ZY05719_08280	AKG40974.1	Cell division protein FtsI
Regulation of transcription	ZY05719_00040	AKG39466.1	Transcription-repair coupling factor
	ZY05719_09670	AKG41241.1	MarR family transcriptional regulator
	ZY05719_10370	AKG41373.1	Transcriptional regulator
Cell wall	ZY05719_08905	AKG41095.1	Undecaprenyl pyrophosphate phosphatase
	ZY05719_09960	AKG41297.1	Cell wall anchor domain-containing protein
Transmembrane transport	ZY05719_10270; *cbiO*	AKG41357.1	Cobalt transporter ATP-binding subunit
Others	ZY05719_00195	AKG39476.1	Hypothetical protein
	ZY05719_05860	AKG40513.1	Hypothetical protein
	ZY05719_08975	AKG41108.1	Metal-sulfur cluster biosynthetic protein

**TABLE 2:** Genes specifically transcribed under *in vivo* biofilm conditions of S. suis captured by SCOTS

Category	Locus tag	Protein accession no.	Putative function
Carbohydrate metabolism	ZY05719_00900; *pgk*	AKG39593.1	Phosphoglycerate kinase
	ZY05719_02230	AKG39831.1	Phosphotransferase system
	ZY05719_07635	AKG40853.1	HPr kinase/phosphorylase
	ZY05719_09080	AKG41128.1	Glycosyl hydrolase-related protein
	ZY05719_01910	AKG39782.1	4-α-Glucanotransferase
Amino acid metabolism	ZY05719_10215	AKG41346.1	tRNA uridine 5-carboxymethylaminomethyl modification enzyme GidA
Nucleotide metabolism	ZY05719_03265	AKG40028.1	Exonuclease III/exodeoxyribonuclease
Amino sugar and nucleotide sugar metabolism	ZY05719_02855	AKG39951.1	CMP-*N*-acetylneuraminic acid synthetase
Proteolysis	ZY05719_07365	AKG40801.1	ATP-dependent Clp protease proteolytic subunit
Protein biosynthesis	ZY05719_01410; *hisS*	AKG39691.1	Histidyl-tRNA synthetase
Nucleotide synthesis	ZY05719_04205; *guaA*	AKG40200.1	GMP synthase
	ZY05719_04870	AKG40321.1	Uracil-DNA glycosylase
	ZY05719_00255	AKG39488.1	Phosphoribosylamine-glycine ligase
Capsular polysaccharide synthesis	ZY05719_02760	AKG39593.1	Cps2C
Cell wall	ZY05719_09495	AKG41206.1	Phosphoglycerol transferase/alkaline phosphatase superfamily protein
Nucleotide excision repair	ZY05719_04965	AKG40339.1	Helicase subunit of the DNA excision repair complex
Restriction modification	ZY05719_03385	AKG41378.1	Type I restriction modification protein HsdS
Cell replication and division	ZY05719_02050	AKG39808.1	Primosome assembly protein PriA
	ZY05719_02365	AKG39858.1	Cell division protein FtsA
Carbohydrate transport	ZY05719_01030	AKG39619.1	Phosphotransferase system, galactitol-specific IIB component
Protein transport	ZY05719_02345	AKG39854.1	Sortase (surface protein transpeptidase)
	ZY05719_08610; *secA*	AKG41039.1	Preprotein translocase subunit SecA
Transmembrane transport	ZY05719_02690	AKG39922.1	Amino acid ABC transporter permease
	ZY05719_02700	AKG39924.1	Glutamine ABC transporter substrate-binding protein
Signal transduction	ZY05719_06490	AKG40636.1	Signal transduction histidine kinase
	ZY05719_02070	AKG39812.1	Serine/threonine protein kinase
Others	ZY05719_09440	AKG41197.1	Aminoglycoside phosphotransferase
	ZY05719_02625	AKG39909.1	Transposase
	ZY05719_06820	AKG40697.1	Large-conductance mechanosensitive channel
	ZY05719_01885	AKG39777.1	ATP-binding protein
	ZY05719_03865	AKG40134.1	Xylanase/chitin deacetylase

**TABLE 3:** Genes specifically transcribed under *in vitro* biofilm conditions of S. suis captured by SCOTS

Category	Locus tag	Protein accession no.	Putative function
Carbohydrate metabolism	ZY05719_04250	AKG40208.1	Phosphomannomutase
	ZY05719_05605	AKG40463.1	UDP-*N*-acetylglucosamine 1-carboxyvinyltransferase
	ZY05719_02660	AKG39916.1	Pyruvate kinase
	ZY05719_02220	AKG39829.1	β-Galactosidase
Amino acid metabolism	ZY05719_03035	AKG39983.1	Arginine deiminase
Nucleotide metabolism	ZY05719_02335	AKG39852.1	Ribonucleases G and E
Energy metabolism	ZY05719_05235	AKG40391.1	Pantothenate kinase
Proteolysis	ZY05719_06615	AKG40661.1	Amylase-binding protein B
Amino acid synthesis	ZY05719_04095; *glyA*	AKG40178.1	Serine hydroxymethyltransferase
Protein biosynthesis	ZY05719_04785; *rplL*	AKG40304.1	50S ribosomal protein L7/L12
	ZY05719_09175; *rpsO*	AKG41146.1	30S ribosomal protein S15
	ZY05719_02270; *valS*	AKG39839.1	Valyl-tRNA synthetase
	ZY05719_08385	AKG40995.1	Glycyl-tRNA synthetase subunit β
Nucleotide synthesis	ZY05719_09920	AKG41289.1	GTP pyrophosphokinase
	ZY05719_00365	AKG39505.1	Folylpolyglutamate synthase
	ZY05719_07765	AKG40877.1	Uridine kinase
Ribosome biogenesis	ZY05719_02055	AKG39809.1	tRNA and rRNA cytosine-C5-methylase
Cellular redox progression	ZY05719_01100	AKG39632.1	NADH-flavin reductase
Cell replication and division	ZY05719_07745	AKG40874.1	DNA polymerase III subunits gamma and tau
	ZY05719_03700	AKG40105.1	DNA topoisomerase IV subunit A
Cell surface protein	ZY05719_01065	AKG39626.1	Cell surface protein
	ZY05719_06545	AKG40647.1	Cell surface protein
	ZY05719_06925	AKG40717.1	Flotillin
Cell wall	ZY05719_09420	AKG41193.1	Penicillin-binding protein
	ZY05719_03880; *murB*	AKG40137.1	UDP-*N*-acetylenolpyruvoylglucosamine reductase
Transmembrane transport	ZY05719_03890	AKG40139.1	Spermidine/putrescine ABC transporter permease I
	ZY05719_03235	AKG40022.1	MFS transporter
	ZY05719_09375	AKG41185.1	Bacterocin transport accessory protein, Bta
Transcriptional regulation	ZY05719_08635	AKG41044.1	LacI family transcriptional regulator
	ZY05719_01875	AKG39775.1	MerR family transcriptional regulator
Signal transduction	ZY05719_07905	AKG40901.1	Two-component regulator, chemotaxis protein CheY
Virulence factors	ZY05719_06690	AKG40676.1	Hemolysin
Others	ZY05719_00345	AKG39501.1	HAD family hydrolase
	ZY05719_06960	AKG40724.1	Hypothetical protein
	ZY05719_00840	AKG39581.1	Hypothetical protein
	ZY05719_00985	AKG39610.1	Extracellular protein

### Analysis of RNA sequencing results and validation of RNA sequencing and SCOTS results by reverse transcription-quantitative PCR (qRT-PCR).

RNA sequencing results showed that there were significant differences in gene expression between the planktonic and biofilm cells *in vitro*. There were 2,254 genes in the transcriptome data, among which 1,470 genes showed significant changes in the transcription process compared with that in the planktonic bacteria (log_2_ fold change [FC] of >0 and *q* value of <0.05). Comparing the genes captured by SCOTS under *in vitro* biofilm conditions, we found that 63 of 69 genes (91.30%) were matched with differentially expressed genes in the *in vitro* biofilm transcriptomic data, with the exceptions being ZY05719_02660, ZY05719_02335, ZY05719_06545, ZY05719_03880, ZY05719_09920, and ZY05719_03235, which to a certain extent verifies the reliability of SCOTS technology in the screening of gene expression differences under specific conditions (Table S2). In addition, about 8.7% of genes could not be detected by RNA sequencing, confirming an important feature of this technology, which is that SCOTS has advantages in analyzing low-abundance genes. It can be seen that SCOTS technology combined with RNA sequencing is an effective method to explore bacterial transcriptomes *in vivo* or under specific circumstances. Furthermore, genes specifically expressed under the *in vivo* biofilm condition by captured SCOTS do not overlap with the valid data obtained by *in vitro* transcriptomics, which indicates that there are indeed differences in gene expression when bacteria form biofilms *in vivo* and *in vitro*. It follows that SCOTS is indeed a reliable method to capture genes specifically expressed in the process of bacterial biofilm formation *in vivo*.

**FIG 6:**
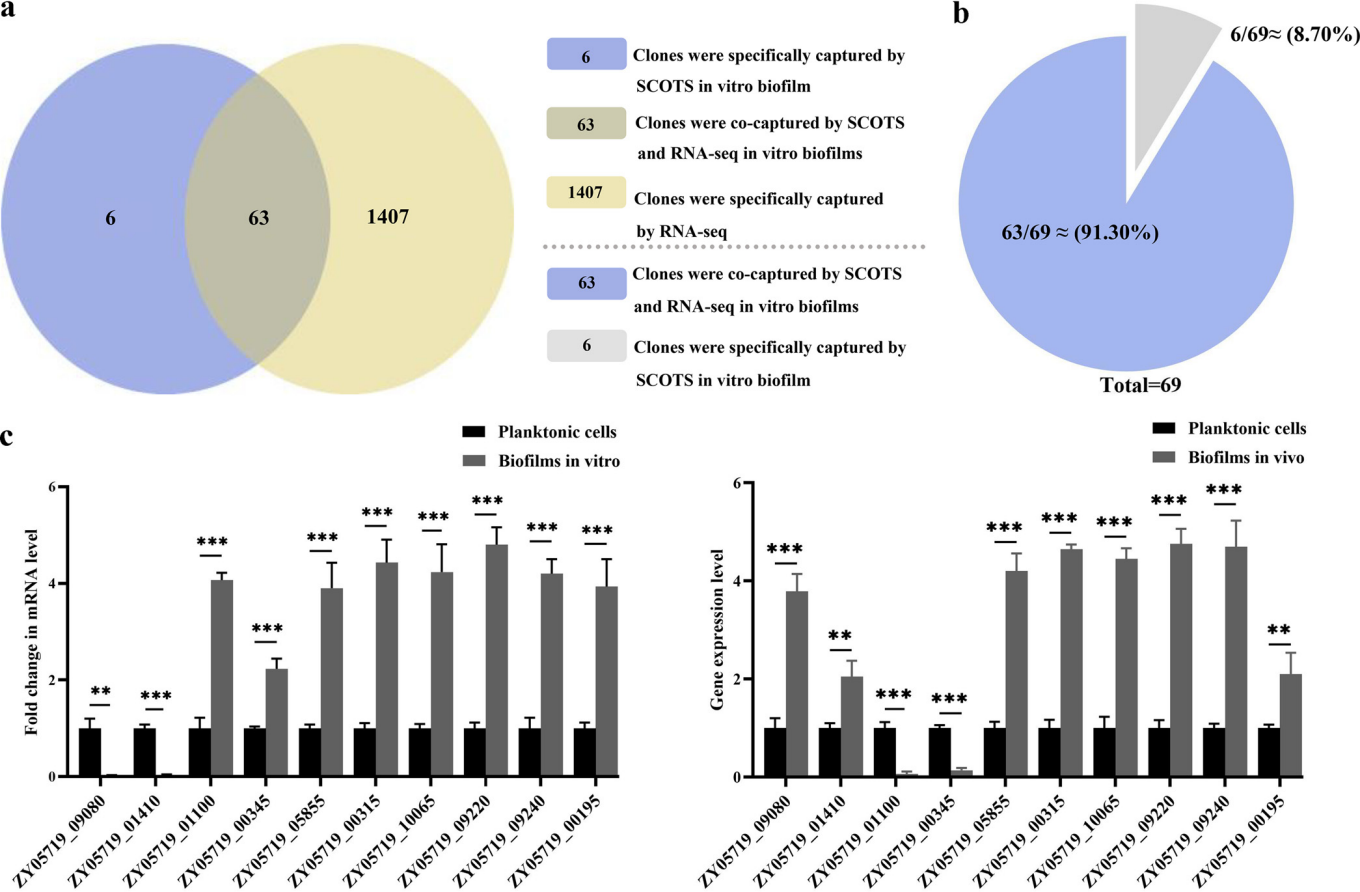
Reliability analysis of differentially expressed genes captured by SCOTS. (a) SCOTS combined with RNA sequencing to screen the number of differentially expressed genes in *in vitro* biofilms. (b) In all, 8.7% of genes were screened by SCOTS but not by RNA sequencing. (c) Expression of genes captured by SCOTS in *in vitro* and *in vivo* biofilm. Left, *in vitro* biofilm group; right, *in vivo* biofilm group. Error bars show standard deviations. *, a *P* value of <0.05 was considered to be significant; **, a *P* value of <0.01 was considered to be very significant; ***, a *P* value of <0.001 was considered to be extremely significant.

To validate the SCOTS-based technology and RNA sequencing results, qRT-PCR was used to detect the differential expression between biofilms and planktonic cells of 10 randomly selected genes. The results for gene expression levels of the *in vivo* biofilm group and the *in vitro* biofilm group are shown in Fig. 6. The expression levels of 8 genes were upregulated to varying degrees in biofilms *in vitro* compared with their levels in the planktonic cells, except for ZY05719_09080 and ZY05719_01410, which were classified as specifically expressed in *in vivo* biofilm in the SCOTS results. It can be seen that the qRT-PCR results are consistent with the data captured by RNA sequencing and SCOTS technology. qRT-PCR was also used to detect the expression of these genes in the *in vivo* biofilm group, and the results showed that the expression of ZY05719_01100 and ZY05719_00345, classified as specifically expressed in *in vitro* biofilm in the SCOTS results, were not upregulated, which is consistent with the SCOTS results (Fig. 6).

## DISCUSSION

According to the National Institutes of Health, approximately 80% of recurrent microbial infections are associated with biofilms, and most clinical cases of persistent infections are attributed to the formation of microbial biofilms (10). S. suis is a commensal of the swine respiratory tract, particularly the tonsils and nasal microbiota, and horizontal transmission through the respiratory tract caused by nose-to-nose contact between swine populations is the predominant route of transmission under natural conditions. To explore the formation mechanisms of biofilm *in vivo* by S. suis, we simulated the natural route of infection using weaning piglets, which are susceptible animals for S. suis. An *in vivo* model of S. suis biofilm adhered to the host mucosa or soft tissue was constructed by nasal inoculation of pigs. Based on the clinical findings, corroborated with histopathological observations, a diagnosis of S. suis infection was made, and subsequently, lung tissues were selected to detect the presence of the *in vivo* biofilm. Up to now, it appears that there is no universal standard for the detection of *in vivo* biofilms. In fact, the methods used to detect *in vivo* biofilms are always challenging. In general, the *in vivo* biofilm detection methods are mainly categorized as either microscopic methods or nonmicroscopic methods. H&E staining, Gram staining, scanning electron microscopy, immunohistochemistry, and immunofluorescence can detect the presence of biofilms *in vivo* under microscopic conditions, while CFU measurement bacterial-count calculation, whole-animal imaging, and detection of biofilm-specific antibodies under nonmicroscopic conditions can be used to confirm the presence of biofilms (8, 15, 22–24).

In this study, microscopic and nonmicroscopic methods were combined to explore the *in vivo* biofilm of S. suis. We used CFU to measure bacterial counts to calculate the numbers of bacteria in organs, and then we used H&E staining to preliminarily detect biofilms. H&E staining has been identified as an accurate predictor for identifying the presence or absence of biofilms *in vivo* and has been used in numerous studies (25–27). In accordance with previous findings, we detected basophilic aggregates of various sizes and morphologies in lung tissues from infected pigs (27). To clarify whether basophilic aggregates in tissues were formed by S. suis or other bacteria, immunohistochemistry and immunofluorescence were used to further detect the presence of biofilms of S. suis *in vivo*, taking advantage of the properties of antibody-specific antigen binding. Biofilms have been defined as aggregates of microorganisms in which cells are frequently embedded in a self-produced EPS. In view of this, aggregated bacteria detected in this study should be considered biofilms of S. suis *in vivo*. Based on our results presented above, it is obvious that biofilms of S. suis *in vivo* detected in this study were mainly distributed in the conductive zone of lung tissues, similar to a previous study on Pseudomonas aeruginosa (26). The longest and shortest axes of the approximate diameters of biofilm aggregates detected in this study were measured (Table 4). In the host organism, biofilm formation is affected by the nutrient environment in the microenvironment, the host tissue structure, and the immune system. Therefore, compared to those of *in vitro* biofilms grown under artificial culture conditions, the sizes and morphologies of *in vivo* biofilms are significantly different. In contrast to a large-size *in vitro* biofilm attached to a uniform abiotic surface, the size of the *in vivo* biofilm is limited. As far as the results of this study are concerned, the size of the detectable biofilms varied greatly, with the longest axis reaching 201 μm and the shortest axis diameter as small as 6 μm, which is consistent with the statistical results of Bjarnsholt et al. (8). As can be observed in the images in Fig. 1, a disintegration phenomenon appears to be exhibited in some of the larger biofilms, which is accompanied by the observation of smaller biofilms or scattered bacteria in the surrounding tissues. Dispersion is often described as the terminal stage of biofilm formation in traditional *in vitro* biofilm studies. Whether the phenomenon we observe in the images shown in Fig. 1 can be identified as biofilm dispersion remains to be further explored in the future, as it cannot be ruled out that the phenomenon was caused by mechanical force during the slicing process. Another phenomenon was noted wherein occasional inflammatory cell infiltration occurred at the site of biofilm formation, and such results coincided with previous studies reporting that bacteria in the biofilm state were far less able to elicit host immune responses than those in the planktonic state (28). SEM to visualize the surface structure of biofilms adhering to the host tissue was consistent with previous studies in which mushroom-like structures were not observed and structures resembling cobwebs, which are structures presented by the EPS after dehydration, were observed (29).

**TABLE 4:** Biofilm sizes in lung tissue in piglets chronically infected with Streptococcus suis

Visualization method	Approx diam (μm)	Figure(s)
Light microscopy (H&E staining)	~25.7–68	Fig. 1a
	~17–22	Fig. 1a
	~110–142	Fig. 1b
	~100–201	Fig. 1b
	~88–60	Fig. 1b
	~57–84	Fig. 1b
	~88–185	Fig. 1b
	~20–25	Fig. 1b
	~64–141	Fig. 1b
	~11–25	Fig. 1b
	~25–67	Fig. 1b
	~34–45	Fig. 1b
	~6–9	Fig. 1b

Scanning electron microscope	~6–9	Fig. 2b
	~20–25	Fig. 2c
	~15–20	Fig. 2d
	~8–17	Fig. 2ef
	~35–45	Fig. 2g

Immunohistochemistry	~9–16	Fig. 3b
	~16–25	Fig. 3c
	~10–18	Fig. 3d
	~25–65	Fig. 3e
	~23–37	Fig. 3f

Fluorescence microscopy	~8–10	Fig. 4b
	~42–50	Fig. 4b
	~50–100	Fig. 4b

To further explore the formation mechanisms of S. suis *in vivo* biofilm and then explore the differences between *in vivo* and *in vitro* biofilm formation mechanisms, we used SCOTS to screen the differential gene expression profiles of S. suis biofilms both *in vivo* and *in vitro*. To our knowledge, this is the first study to apply the SCOTS method to the screening of preferentially expressed genes in both *in vivo* and *in vitro* biofilms. To validate the reliability of SCOTS, we performed RNA sequencing of *in vitro* biofilm samples and alignment impressions with the genes screened by SCOTS, which confirmed the reliability of SCOTS and identified some low-abundance transcripts that were not captured by RNA sequencing. Specifically, in this study, we used SCOTS to screen 64 and 69 genes specifically transcribed in biofilms *in vivo* and *in vitro*, respectively. These genes are mainly concentrated in bacterial metabolic processes, which is not surprising; in fact, a large number of studies have pointed out that bacteria growing in a biofilm state show physiological and metabolic differences from their planktonic counterparts (30, 31).

Significant differences between *in vivo* and *in vitro* gene expression profiles have been reported for bacteria, and there is anatomical site specificity in gene expression *in vivo* (28, 32). When it comes to the genes screened in this study both *in vitro* and *in vivo*, 21 of the 33 genes screened are related to bacterial metabolism, but few of these screened genes have been shown to be involved in biofilm formation in previous studies. Among them, ZY05719_09405 encodes a subtilis-like serine protease related to proteolysis, and its homologues in species like Vibrio cholerae and Streptococcus pneumoniae are closely related to the dispersal of bacterial biofilms (33, 34). ZY05719_09670 encodes a transcriptional regulator belonging to the MarR family, which has been shown to be involved in negatively regulating bacterial biofilm formation in a variety of bacteria, including SarZ in Staphylococcus aureus (35), BifR in Burkholderia thailandensis (36), and EspR in Streptococcus mutans (37). Some examples of positive regulation also exist, such as TcaR in S. aureus (38) and HpaR in Yersinia pseudotuberculosis (39). The ZY05719_08905-encoded protein is a homologue of undecaprenyl pyrophosphate phosphatases, related to bacterial cell wall synthesis and confirmed to be involved in the regulation of biofilm in Mycobacterium smegmatis (40). Likewise, the biofilm formation of a *uppP* transposon mutant strain of S. mutans was defective (41). Except for the 3 genes mentioned above, most of the gene homologues related to biofilm formation in other bacteria have not been reported. In addition to the genes coexpressed in the biofilm state *in vitro* and *in vivo*, genes captured specifically in the biofilm state *in vivo* deserve our attention, since these genes are not detected under the biofilm state *in vitro* and are often ignored. The functional classification of these genes also focuses on bacterial metabolism. Among them, the carbohydrate metabolism-related gene *pgk* (ZY05719_00900) was identified in the previous comparative proteomic analysis of the immunogenic proteins related to S. suis biofilm infection (5). In addition, the proteolysis-related ATP-dependent Clp protease proteolytic subunit ClpP, encoded by ZY05719_07365, has been confirmed to affect S. suis biofilm formation, and the deletion of *clpP* significantly weakened the ability of S. suis to form biofilm. Researchers reasoned that the significant defect in biofilm formation might be attributable to alterations in the bacterial cell structure, given the tendency of the *clpP* deletion strains to form longer chains (42). ZY05719_03385, which is related to the restriction modification of S. suis, encodes the type I restriction modification protein HsdS, which was proved to contribute to the survival ability of S. suis in phagocytes and was first shown to correlate with S. suis biofilm formation (43). ZY05719_02365 encodes the cell division protein FtsA, which is associated with cell replication and division; its homologue in Enterococcus faecalis is specifically overexpressed 2-fold under biofilm conditions (44). Homologues of transport-related sortases like that encoded by ZY05719_02345 were confirmed to be involved in the biofilm formation of S. aureus, S. mutans, and Streptococcus gordonii (45). Two-component systems comprised of serine threonine kinase protein kinases and phosphatases are often used by bacteria to detect, transmit, and respond to external signals. The signal transduction histidine kinase and the serine/threonine protein kinase are encoded by ZY05719_06490 and ZY05719_02070, respectively. Their homologs in other bacteria have been shown to be associated with biofilm formation. A previous study pointed out that in S. epidermidis, the two-component system YycG/YycF is crucial in biofilm formation and that monoclonal antibodies to these proteins can specifically inhibit the formation of biofilm (46). Much evidence exists for the association of serine/threonine kinases with bacterial biofilms. Examples include PknB in S. mutans (47), PknF in Mycobacterium tuberculosis (48), Stk in S. epidermidis, and PpkA in P. aeruginosa (49, 50). Among the genes captured by SCOTS that are specifically expressed in biofilms *in vitro*, many genes overlapped with previous studies on S. suis biofilm. Arginine deiminase, amylase-binding protein B, UDP-*N*-acetylglucosamine 1-carboxyvinyltransferase, etc., have been confirmed to be related to S. suis biofilm formation in previous studies, which is also reliably confirmed by the SCOTS technology.

Taken together, we can clearly find that there are great differences in the gene expression profiles of S. suis in the *in vivo* and *in vitro* biofilm states, especially in metabolism-related genes. It follows that the study of biofilms under different conditions cannot simply be generalized. Our study provides a list of candidate genes potentially involved in S. suis biofilm formation, which will facilitate further exploration of chronic infections caused by biofilms of S. suis. Future studies should focus on those genes that are coexpressed in *in vitro* and *in vivo* biofilms, as well as genes specifically expressed in *in vivo* biofilms. However, these screened genes can be used as a reference only and cannot be inferred to be important for biofilm formation solely because of the expression of a certain gene.

## MATERIALS AND METHODS

### Bacterial strains, plasmids, primers, culture conditions, and experimental animals.

The virulent S. suis strain ZY05719 of serotype 2 was used in this study. This strain was isolated in Ziyang, China, in 2005 and has been confirmed to form biofilms *in vitro*. Escherichia coli DH5α was used as the host strain for plasmid construction, replication, and preservation. The plasmid pMD19-T vector was used as the source for rRNA genes (16S rRNA and 23S rRNA), and SCOTS clones were prepared in pMD19-T. S. suis was cultured on Todd-Hewitt broth (THB) (Oxoid Ltd., Basingstoke, UK) or on Todd-Hewitt agar (THA) at 37°C. E. coli DH5α was cultured routinely in Luria-Bertani broth or plates (Oxoid, Basingstoke, UK) at 37°C. Ampicillin (50 μg/mL), IPTG (isopropyl β-d-thiogalactopyranoside) (100 μg/mL), and X-Gal (5-bromo-4-chloro-3-indolyl-β-d-galactopyranoside) (200 μg/mL) in LB medium were used as required. All primers used in this study are shown in Table S1. Healthy, S. suis-free, 30-day-old female piglets were obtained from a farm free of relevant swine pathogens. Piglets were randomly divided into two groups: uninfected (*n* = 1) and infected (*n* = 5).

### *In vitro* biofilm formation on polystyrene tissue culture plates.

The *in vitro* biofilm formation assay for S. suis was performed on 6-well polystyrene tissue culture plates as described previously. A detailed procedure for *in vitro* biofilm culture can be found in our previously published manuscript (2).

### Establishment of an *in vivo* biofilm model of S. suis: animal infections and determination of bacterial load in tissues.

Three 30-day-old S. suis-free piglets were inoculated intranasally with 1 mL bacterial suspension containing 1 × 10^8^ CFU of S. suis ZY05719. After infection, the piglets were monitored daily for clinical symptoms of illness. Piglets in the infected group began to show most of the typical symptoms successively, such as depression, elevated body temperature, joint swelling, and dyspnea, approximately 36 h after inoculation. Slaughter was performed at 72 h after infection, when all piglets in the infected group showed typical clinical signs. Tissue samples (lungs, tonsils, liver, heart, spleen, and brain) were aseptically collected, weighed, and homogenized. The animals were housed and handled in accordance with the Laboratory Animal Guidelines for Ethical Review of Animal Welfare of China (GB/T 35892-2018). Serial 10-fold dilutions of tissue homogenates were made with phosphate-buffered saline (PBS) (1 mL/g) and plated on THA agar plates for colony counting. The tissue type with the highest bacterial loads was used in the subsequent experiments.

### H&E staining.

H&E staining has been widely used as a traditional tissue staining method to detect histopathological changes and has similarly been shown to detect biofilms *in vivo*.

H&E staining was performed referring to the previously published method (51). After that, pathological tissue sections were placed for imaging in a full-field digital slice scanner (Pannoramic Midi, Hungary) at ×20 magnification.

### SEM of biofilms.

For scanning electron microscopy (SEM), tissue samples were processed according to a protocol previously published by our group (2). SEM images of the tissue samples were obtained at 20 kV accelerating voltage by using a field emission scanning electron microscope (JSM-5610LV; JEOL, Japan).

### Immunohistochemistry.

The previously prepared paraffin sections were deparaffinized. First, the paraffin sections were baked at 55°C for 24 h, and then they were deparaffinized twice for 5 min in xylene. Slides were rehydrated with decreasing ethanol concentrations (ranging from 100% to 95%, 70%, and 50%), followed by washing with distilled water and 0.05 M PBS (pH 7.1). H_2_O_2_ at 3% was used to incubate the samples for 10 min at room temperature to block endogenous peroxidase activity in the tissue samples. Slides were microwaved for 10 min in sodium citrate buffer (pH 6.0) to recover antigen, and then endogenous peroxidase activity was blocked by using peroxidase blocking solution after slides were cooled down to room temperature naturally. Slides rinsed in PBS were incubated with nonimmunized healthy pig serum for 10 min at room temperature, followed by a PBS rinse. Then, serum from pigs that had recovered from infection with S. suis was added to the slides and the slides were incubated at room temperature for 1 h, washed with PBS, treated with HRP-labeled rabbit anti-pig IgG, incubated at room temperature for 1 h, and rinsed with PBS again. Approximately 100 μL of diaminobenzidine (DAB) liquid was subsequently added to the slides for 10 min to detect antibody binding, after which the slides were rinsed with distilled water at room temperature to stop the reaction. Finally, the slides were stained with hematoxylin and differentiated with 1% hydrochloric acid and ethanol, followed by mounting in neutral resin. Similarly, the slices were scanned by a fully automated slide scanner as mentioned above.

### Immunofluorescence.

For the immunofluorescence assay, the initial steps up to adding the secondary antibody were similar to those mentioned above in “Immunohistochemistry,” but the secondary antibody used for the immunofluorescence assay was fluorescein isothiocyanate (FITC)-labeled rabbit anti-pig IgG, which is different from the HRP-labeled rabbit anti-pig IgG used for immunohistochemistry. After incubating with the secondary antibody for 1 h, PBS was added to the slides and the slides sealed with neutral resin. Photomicrographs were captured under an Axio Observer A1 inverted fluorescence microscope. FITC-labeled antibody in the tissue sections was measured by using an inverted fluorescence microscope (Axio Observer A1; Zeiss) fitted with an FITC filter set under dark-field illumination through a low numerical aperture (NA) (NA = 0.35, ×100 magnification) and a high NA (NA = 0.8, ×400 magnification).

### Preparation of genomic DNA and PCR cloning of 16S and 23S rRNA genes of S. suis.

Genomic DNA (gDNA) of S. suis was extracted using the PureLink genomic DNA minikit (Thermo Fisher Scientific) and subsequently tagged using biotin. gDNA was used as a template, and primer pair 16S01/-02 was used to amplify the bacterial 16S rRNA gene, while primer pairs 23SC01/-02 and 23SN01/-02 were used to amplify the C terminus and N terminus, respectively, of the 23S rRNA gene. PCR amplification products were cloned into the pMD19-T vector (TaKaRa, Japan), and the plasmids were introduced into E. coli DH5α in accordance with the manufacturer’s protocol. The biotinylated S. suis gDNA (12 μg) was then mixed with pMD19-rRNA gene plasmids (16S rRNA, 23S1 rRNA, and 23S2 rRNA genes, 1:1:1) and sonicated for 10 min. After sonication, the mixture was precipitated with sodium acetate (NaAc) and absolute ethanol, washed with 75% ethanol, dissolved with EDTA, and then stored at −20°C after being aliquoted.

### RNA isolation and cDNA synthesis and amplification.

For *in vivo* biofilm, amounts of about 0.1 g of the tissue samples with the highest bacterial loads were taken immediately after necropsy and stored in prechilled mortars, and the tissue samples were ground to powder in liquid nitrogen. For *in vitro* biofilm, in order to exclude an effect of planktonic bacteria on the experimental results, after the 6-well polystyrene tissue culture plates containing the S. suis bacteria were cultured at 37°C for 24 h, the supernatant in the wells was removed using a pipette and the wells washed twice with distilled water, followed by scraping and collecting biofilm off the plates. For planktonic bacteria, when the optical density at 600 nm (OD_600_) of the planktonic S. suis bacteria cultured under shaking conditions reached 0.6 to 0.8, the bacterial solution was rapidly precooled and the precipitant was collected and then used for RNA isolation. Total RNAs from *in vivo* biofilm (S. suis-infected tissues), *in vitro* biofilm, and planktonic bacteria were isolated using TRIzol reagent according to the manufacturer’s instructions. RNA samples isolated from three different sources extracted from the above-described steps were analyzed by *A*_260_/*A*_280_ spectrophotometer readings and electrophoresis, respectively. Three RNAs extracted from *in vivo* and *in vitro* biofilm samples of S. suis and six RNAs extracted from S. suis cultured in normal THB medium were taken for cDNA synthesis. The first strand of cDNA was synthesized using Moloney murine leukemia virus (M-MuLV) reverse transcriptase (RNase H) and random hexamer primer. Primer SCOTSN601 was used to amplify the total RNA of bacteria cultured normally in THB medium, while primer SCOTSN602 was used to amplify the RNA of the *in vitro* and *in vivo* S. suis biofilm samples. The reaction conditions for first-strand synthesis were 5 min at 25°C and 1 h at 42°C, followed by 70°C for 15 min. After that, the second-strand synthesis was carried out with Klenow fragment. cDNA was then amplified by PCR by using the defined primers for each set of cDNA for 25 cycles. The constructed cDNA libraries were amplified using primer SCOTS01 or SCOTS02 under the following conditions: 94°C for 5 min, 35 cycles of 94°C for 30 s, 58°C for 45 s, and 72°C for 1 min, and 72°C for 20 min. Specifically, SCOTS01 primer was used to obtain the cDNA of normal culture condition in THB medium cultured normally *in vitro*, while SCOTS02 was used to amplify the cDNA of biofilm state bacteria *in vitro* and vivo. Double-stranded cDNA was purified and recovered using the E.Z.N.A. cycle pure kit (Omega).

### Selective capture of transcribed sequences (SCOTS).

Six sonication mixtures of biotin-labeled S. suis gDNA and rRNA gene plasmids were predenatured at 98°C for 3 min, followed by the addition of 2 μL of 1 mol/L NaCl solution for a 30-min prehybridization at 67°C to preblock rRNA-encoding regions of the genomic DNA, thereby rendering these sites unavailable for hybridization with rRNA genes present in the cDNA mixtures. PCR-amplified products (5 μg, 8 μL) of cDNA libraries constructed from bacteria grown under normal culture conditions in THB medium and biofilms *in vivo* were predenatured by coincubation at 98°C for 3 min. The same treatment method was then applied to prehybridize PCR-amplified products of cDNA libraries constructed from bacteria grown under normal culture conditions in THB medium and biofilms *in vitro*. For each group, 3 parallel repetitions were set. After prehybridization, S. suis gDNA that had been prehybridized with rRNA gene plasmids was added and hybridized for 24 h at 68°C. Streptavidin-coated magnetic beads were used to capture cDNAs according to the manufacturer’s instructions. After elution and precipitation, the cDNAs were reamplified by PCR using primer SCOTS01 or SCOTS02. The amplified cDNA products were purified and recycled, and the next round of SCOTS was performed, including prehybridization, hybridization, capture, and PCR amplification enrichment. After three rounds of SCOTS, cDNAs of S. suis under each growth condition were finally obtained for cloning into the pMD19-T vector in E. coli DH5α. SCOTS libraries of S. suis genes differentially transcribed under *in vitro* and *in vivo* biofilm conditions were individually constructed for dot blot hybridization, as well as subsequent sequencing detection.

### Southern dot blot.

The single clones in the SCOTS libraries of S. suis genes differentially transcribed under *in vitro* and *in vivo* biofilm conditions constructed in the previous step were checked using the SCOTS02 primer, and the PCR products with only single bands were taken for subsequent dot hybridization. cDNA libraries after 3 rounds of SCOTS, including the libraries from the *in vivo* biofilm state, the *in vitro* biofilm state, and the THB culture state, were mixed with DIG-High Prime to prepare the probes for dot hybridization. Then, 2-μL amounts of PCR products of positive clones from the *in vivo* biofilm or *in vitro* biofilm were mixed with 2 μL NaOH (0.6 M) to denature, and 1-μL amounts of the mixtures were placed on the same positions of the two nylon membranes. The nylon membranes were then baked at 120°C for 30 min in order to transfer and fix the DNA. Subsequently, two denatured probes (*in vitro* biofilm probe combined with THB culture state probe or *in vivo* biofilm probe combined with THB culture state probe) were added to the nylon membrane and hybridized overnight at 42°C. After 4 washes and blocking, anti-digoxigenin antibody was added. After 2 washes and equilibration, the nylon membranes were placed in the color substrate solution and reacted in the dark until the color appeared, and then Tris-EDTA (TE) buffer was added to stop the color reaction. Two consecutive rounds of dot blot validation were performed to eliminate false-positive results. After two rounds of dot hybridization, positive clones were screened.

### RNA sequencing and validation of SCOTS results by qRT-PCR.

To verify the reliability of SCOTS detection, RNA sequencing was also used to determine the distinct transcriptional profiles of planktonic and biofilm cells. Given the characteristics of biofilms adhering to visceral tissues *in vivo*, the inability to circumvent RNA isolation from host tissue cells during RNA extraction of biofilms *in vivo*, and the low amounts of pathogen RNA relative to the host RNA background, this poses a major challenge for RNA sequencing of *in vivo* biofilms. Therefore, only RNA sequencing analysis of *in vitro* biofilm was performed in this study. RNA was extracted from planktonic cells grown for 8 h and from S. suis biofilms formed after 24 h by using the RNeasy minikit following the manufacturer’s protocol. After RNase-free DNase I treatment, the purity of the RNAs was detected by agarose gel electrophoresis, and the concentration and integrity of the RNAs were determined by using the NanoDrop 1000 spectrophotometer (Thermo Fisher Scientific, USA). The extracted RNA was stored at −80°C, and then all samples were sent to Novogene Bioinformatics Technology (Beijing, China) for library construction and sequencing with an Illumina HiSeq 2500 sequencer.

The mapping was performed with reference to the S. suis strain 05ZYH33 genome. Gene expression profiles obtained by RNA sequencing were compared with genes expressed under *in vitro* biofilm conditions captured by the SCOTS method to test the reliability of the SCOTS technology. Subsequently, according to the RNA extraction method described above, the cDNAs of three groups of bacteria (planktonic cells, *in vitro* biofilms, and *in vivo* biofilms) were extracted, and the SYBR green fluorescence quantitative PCR method was used to detect the genes screened by the SCOTS method. Specifically, 6 genes expressed in both *in vivo* and *in vitro* biofilms, 2 genes specifically expressed in *in vivo* biofilms, and 2 genes specifically expressed in *in vitro* biofilms were randomly selected. All primers and sequences are listed in Table S3. Quantitation and melting curve analyses for qRT-PCR were performed using universal SYBR qPCR master mix (100,010,629,049; Biosharp, China) according to the manufacturer’ instructions. The qRT-PCRs were carried out with 3 biological replicates, and each biological replicate was carried out in triplicate per plate. The qRT-PCR conditions were as follows: 95°C for 30 s, followed by 40 cycles at 95°C for 5 s and 60°C for 30 s. 16S rRNA of S. suis was used as the internal reference to normalize the data. The expression levels of these genes in planktonic bacteria were used as controls to detect their expression levels in biofilm bacteria *in vivo* and *in vitro*.

### Statistical analysis.

GraphPad Prism 9.0 software was used to perform statistical analyses for all data. The significance of the data in Fig. 6c was analyzed according to the unpaired Student’s two-sided *t* test, and *P* values are described in the legend.

### Data availability.

The data sets generated and/or analyzed during the current study are available in the NCBI Gene Expression Omnibus (GEO) repository database under the series record (accession number GSE217756; https://www.ncbi.nlm.nih.gov/gds/?term=GSE217756).
