# Supplementary material for: Establishment of Streptococcus suis Biofilm Infection Model In Vivo and Comparative Analysis of Gene Expression Profiles between In Vivo and In Vitro Biofilms
Source: Microbiol Spectr. 2022 Dec 12;11(1):e02686-22. doi: 10.1128/spectrum.02686-22 (PMC9927446; doi:10.1128/spectrum.02686-22)
Supplement: Supplemental file 1 — Supplemental material. Download spectrum.02686-22-s0001.pdf, PDF file, 5.6 MB [file spectrum.02686-22-s0001.pdf]

Supplementary Figure legends

Figure S1. Determination of bacterial load in organs of artificially infected piglets.

Figure S2. Histopathologic sections of uninfected and artificially infected lungs assessed by hematoxylin and eosin staining (x200). Severe pathological changes were observed in the infected group, manifested in alveolar hemorrhage (①), alveolar wall hemorrhage (②), thickening (③), inflammatory cell infiltration (④), alveolar luminal exudate was organized to form fibrous connective tissue(⑤), serous exudate (⑥) and bronchiolar hemorrhage (⑦).

Supplementary Table 1. Primers used for SCOTS screening.

Supplementary Table 2. Genes specifically transcribed under in vitro biofilm conditions in *S. suis* co-identified by SCOTS and RNA sequencing.

Supplementary Table 3. Primers for qRT-PCR used in this study.

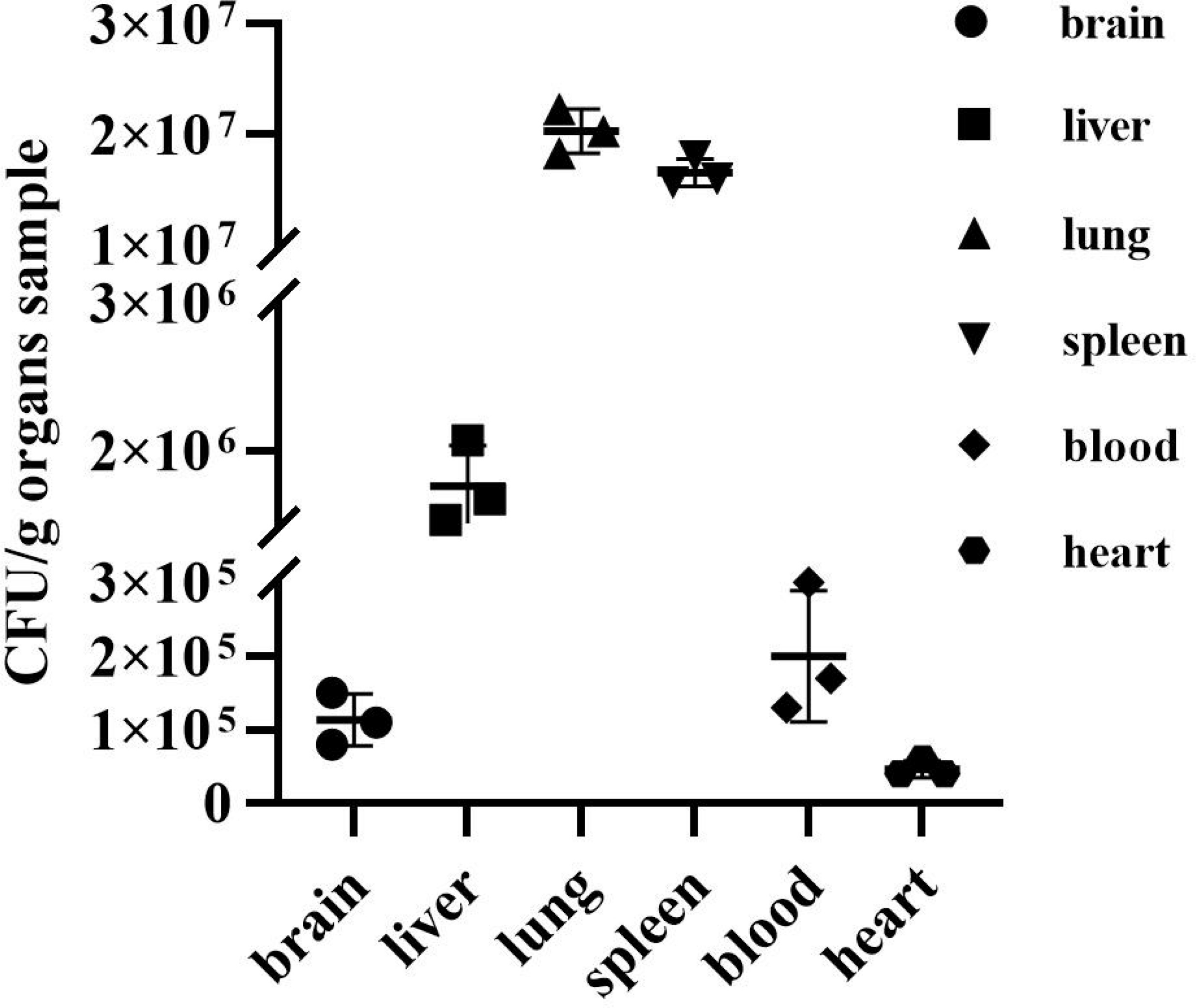
CFU/g organs sample
3×10^7
2×10^7
1×10^7
3×10^6
2×10^6
3×10^5
2×10^5
1×10^5
0
brain
liver
lung
spleen
blood
heart
brain
liver
lung
spleen
blood
heart

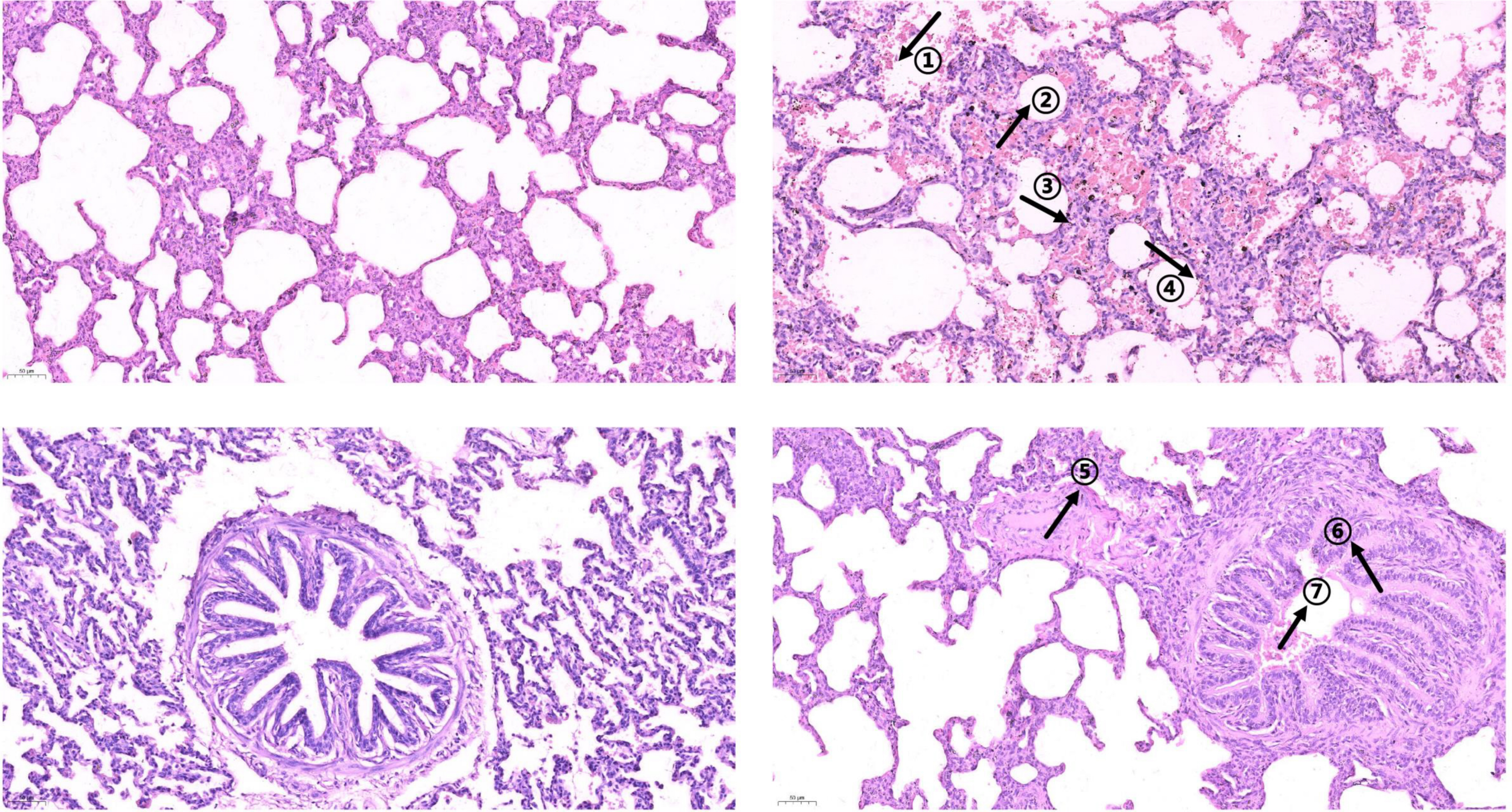

Uninfected group

Infected group

Supplementary Table 1. Primers used for SCOTS screening.

Primers	Sequences
SCOTS-N6-01	5′-GCCGGTCGACTGCAGAATTC-N6-3′
SCOTS-N6-02	5′-CTACGCATGCTCGAGGTACC-N6-3′
SCOTS01	5′-GCCGGTCGACTGCAGAATTC-3′
SCOTS02	5′-CTACGCATGCTCGAGGTACC-3′
16S01	5′-CGTACACTCGAGAATACATGCAAGTGGAACGC-3′
16S02	5′-CGTATCGAGCTCATCATCTATCCCACCTTAGG-3′
23SN01	5′-CGTACAGTCGACTTGGGGAGCTGTAAGTAAGC-3′
23SN02	5′-CGTATCGAGCTCTTGCCGAGTTCCTTAACGAG-3′
23SC01	5′-CGTACAGTCGACTAAGGAACTCGGCAAAATGG-3′
23SC02	5′-CGTATCGAGCTCTGGGAAATCTCATCTTGAGG -3′

Supplementary Table 2. Genes specifically transcribed under in vitro biofilm conditions in *S. suis* co-identified by SCOTS and RNA sequencing.

Category	Locus tag	Protein ID	Putative function	Log_2-FC
Carbohydrate metabolism	ZY05719_00790	AKG39571.1	Type II secretory pathway, pullulanase PulA and related glycosidases	1.3189
	ZY05719_01910	AKG39782.1	4-α -glucanotransferase	-0.98264
	ZY05719_09285	AKG41167.1	Dihydroxyacetone kinase	-1.5922
	ZY05719_03245	AKG40024.1	Phosphoglycerate dehydrogenase	0.37665
	ZY05719_05855	AKG40512.1	Ribokinase family sugar kinase	2.2038
	ZY05719_04250	AKG40208.1	Phosphomannomutase	-1.209
	ZY05719_05605	AKG40463.1	UDP-N-acetylglucosamine1-carboxyvinyltransferase	1.3977
	ZY05719_02660	AKG39916.1	Pyruvate kinase	
	ZY05719_02220	AKG39829.1	β-galactosidase	-1.8738
Amino acid metabolism	ZY05719_09250	AKG41160.1	Leucyl aminopeptidase (aminopeptidase T)	-0.5905
	ZY05719_08880	AKG41090.1	Selenocysteine lyase	-0.40329
	ZY05719_03035	AKG39983.1	arginine deiminase	-3.0334
Nucleotide metabolism	ZY05719_10310	AKG41365.1	Inosine 5'-monophosphate dehydrogenase	0.85552
	ZY05719_02630	AKG39910.1	Adenosine deaminase	0.86979
	ZY05719_01840	None	Histone acetyltransferase HPA2-like acetyltransferase	-1.3199
	ZY05719_02335	AKG39852.1	ribonucleases G and E	
Nucleotide synthesis	ZY05719_09920	AKG41289.1	GTP pyrophosphokinase	
	ZY05719_00365	AKG39505.1	Folylpolyglutamate synthase	0.78396
	ZY05719_07765	AKG40877.1	Uridine kinase	-1.6938
Ribosome biogenesis	ZY05719_02055	AKG39809.1	tRNA and rRNA cytosine-C5-methylase	-0.47377

Energy metabolism	ZY05719_00315	AKG39496.1	Multidrug ABC transporter ATPase	2.4045
	ZY05719_05235	AKG40391.1	Pantothenate kinase	-1.4792
Amino acid biosynthesis	ZY05719_04095; *glyA*	AKG40178.1	serine hydroxymethyltransferase	-1.4216
Protein biosynthesis	ZY05719_00465; *rplV*	AKG39524.1	50S ribosomal protein L22	0.64704
	ZY05719_03945; *trmD*	AKG40148.1	tRNA (guanine-N (1)-)-methyltransferase	-0.73967
	ZY05719_10065; *argS*	AKG41316.1	Arginyl-tRNA synthetase	2.3423
	ZY05719_09220	AKG41154.1	translation factor (SUA5)	2.5087
	ZY05719_09240	AKG41158.1	2-isopropylmalate synthase	2.26
	ZY05719_06080	AKG40557.1	30S ribosomal protein S16	-0.98155
	ZY05719_04785; *rplL*	AKG40304.1	50S ribosomal protein L7/L12	-1.4866
	ZY05719_09175; *rpsO*	AKG41146.1	30S ribosomal protein S15	-0.64988
	ZY05719_02270; *valS*	AKG39839.1	valyl-tRNA synthetase	-1.2504
	ZY05719_08385	AKG40995.1	glycyl-tRNA synthetase subunit β	-1.257
Proteolysis	ZY05719_08140	AKG40947.1	Cysteine aminopeptidase C	-1.3671
	ZY05719_09405	AKG41190.1	Subtilisin-like serine protease	-0.56552
	ZY05719_02485	AKG39882.1	Collagenase-like protease	0.93383
	ZY05719_00870	AKG39587.1	Chaperonin GroEL	-1.1468
	ZY05719_06615	AKG40661.1	Amylase-binding protein B	-2.1424
Cellular redox progression	ZY05719_01100	AKG39632.1	NADH-flavin reductase	2.4432
	ZY05719_00565	AKG39544.1	DNA-directed RNA polymerase subunit α	0.71027
and division	ZY05719_06580	AKG40654.1	DNA polymerase I	-0.51999
	ZY05719_08280	AKG40974.1	Cell division protein FtsI	1.1396
	ZY05719_07745	AKG40874.1	DNA polymerase III subunits γ and tau	0.34242

	ZY05719_03700	AKG40105.1	DNA topoisomerase IV subunit A	-1.0113
Regulation of transcription	ZY05719_00040	AKG39466.1	Transcription-repair coupling factor	0.8932
	ZY05719_09670	AKG41241.1	MarR family transcriptional regulator	1.0814
	ZY05719_10370	AKG41373.1	Transcriptional regulator	-0.68158
	ZY05719_08635	AKG41044.1	LacI family transcriptional regulator	-0.79938
	ZY05719_01875	AKG39775.1	MerR family transcriptional regulator	-0.43446
Cell surface	ZY05719_01065	AKG39626.1	Cell surface protein	-0.86644
	ZY05719_06925	AKG40717.1	flotillin	-1.427
	ZY05719_06545	AKG40647.1	Cell surface protein	
Cell wall	ZY05719_08905	AKG41095.1	Undecaprenyl pyrophosphate phosphatase	0.99083
	ZY05719_09960	AKG41297.1	Cell wall anchor domain-containing protein	1.0803
	ZY05719_09420	AKG41193.1	Penicillin-binding protein	0.66217
	ZY05719_03880; *murB*	AKG40137.1	UDP-N-acetylenolpyruvoylglucosamine reductase	
Transmembrane transport	ZY05719_10270; *cbiO*	AKG41357.1	cobalt transporter ATP-binding subunit	1.4412
	ZY05719_03890	AKG40139.1	Spermidine/putrescine ABC transporter permease I	-0.51326
	ZY05719_03235	AKG40022.1	MFS transporter	
	ZY05719_09375	AKG41185.1	bacterocin transport accessory protein, Bta	-1.5061
	ZY05719_07905	AKG40901.1	Two-component regulator, chemotaxis protein CheY	-0.43446
Virulence factors	ZY05719_06690	AKG40676.1	Hemolysin	-2.2116
Others	ZY05719_00195	AKG39476.1	Hypothetical protein	1.9146
	ZY05719_05860	AKG40513.1	Hypothetical protein	1.6335
	ZY05719_08975	AKG41108.1	Metal-sulfur cluster biosynthetic protein	-0.1922
	ZY05719_00345	AKG39501.1	HAD family hydrolase	1.0927

ZY05719_06960	AKG40724.1	hypothetical protein	1.3326
ZY05719_00840	AKG39581.1	hypothetical protein	0.44224
ZY05719_00985	AKG39610.1	extracellular protein	-0.31417

Supplementary Table 3. Primers for qRT-PCR used in this study.

Primers	Sequences (5'-3')
ZY05719_09080	F: GGACCTGGGTGACTAGGTGTCT
	R: TTTGGGCTGTCTGGCTGAG
ZY05719_01410	F: GACATTCGCCTCCACCTCAA
	R: CCACAGCAACCTTGTCTTCTTTT
ZY05719_01100	F: AGCGTAGCCCAAGAAGTAATC
	R: TTTGGTGCTTGGACACCA
ZY05719_00345	F: GTCCGTCACCATTTCACAGA
	R: AAATACGGACTGGACAAGGAAA
ZY05719_05855	F: GGACAGTAGCCGTATCTCTTT
	R: CAATAGTCTGCATAGGTCGCA
ZY05719_00315	F: TGCAGTTGGACACGACTAAA
	R: TAGGTGTGGCAGTGGAAATC
ZY05719_10065	F: GTCTAGTGACCAAGGACAAGAAA
	R: GAGTAAGGGCGCGTGAAATA
ZY05719_09220	F: GTCTTGTCTGGCTGGATAGATG
	R: CGTGGCAAGGTAGAAGGTATC
ZY05719_09240	F: GTAGTGCTGAGGAGTCGTAAAG
	R: GCGGACGGAGTTGGATTATT
ZY05719_00195	F: AGCAACAAGTAGCCTCAGTATC
	R: CTACACCAACGGTAAGCACA
